# Treatment of proteinuria in dogs with telmisartan: A retrospective study

**DOI:** 10.1111/jvim.16146

**Published:** 2021-05-10

**Authors:** Julie Lecavalier, Lyanne Fifle, Romain Javard

**Affiliations:** ^1^ DMV Veterinary Center Montréal Quebec Canada

**Keywords:** angiotensin receptor blocker, canine, renin‐angiotensin‐aldosterone system, urine protein : creatinine ratio

## Abstract

**Background:**

Use of telmisartan for the treatment of proteinuria in dogs has not been thoroughly investigated.

**Hypothesis/Objectives:**

Telmisartan can be effective for the treatment of proteinuria in dogs.

**Animals:**

Forty‐four client‐owned dogs with proteinuria.

**Methods:**

Retrospective study. Dogs diagnosed with clinically relevant proteinuria (nonazotemic dogs with a urine protein‐to‐creatinine ratio [UPC] ≥2 and azotemic dogs with UPC ≥0.5) were separated into 3 groups: telmisartan alone, with benazepril, or with mycophenolate. The UPC was recorded before treatment and at subsequent follow‐ups (1, 3, 6, and 12 months, as available). Response to treatment was categorized as complete (UPC ˂0.5), partial (UPC decreased by ≥50% but still ≥0.5), or no response (UPC decreased by <50%). Serum creatinine and potassium concentrations and arterial pressure also were recorded.

**Results:**

In the telmisartan group, treatment response (UPC ˂0.5 or decreased by ≥50%) was observed in 70%, 68%, 80%, and 60% of dogs at 1, 3, 6, and 12 months follow‐up, respectively. No significant changes were noted in serum creatinine or potassium concentrations, or in arterial blood pressure at all follow‐up times. Adverse effects consisted of mild self‐limiting gastrointestinal signs in 5 dogs. Two dogs developed clinically relevant azotemia that required discontinuation of the treatment before the first follow‐up.

**Conclusions and Clinical Importance:**

Telmisartan can be considered for treatment of proteinuria in dogs, alone or in combination with other treatments for proteinuria.

**Abbreviations**
ACEi: angiotensin converting enzyme inhibitors
ACVIM: American College of Veterinary Internal Medicine
ARB: angiotensin receptor blocker
AT1: angiotensin II type 1 receptor
AT2: angiotensin II type 2 receptor
RAAS: renin‐angiotensin‐aldosterone system
SCr: serum creatinine concentration
TOD: target organ damage
UPC: urine protein : creatinine ratio

## 1 INTRODUCTION

Persistent renal proteinuria is associated with increased renal morbidity and increased mortality in dogs and cats.^1^, ^2^, ^3^ The renin‐angiotensin‐aldosterone system (RAAS) regulates blood pressure, fluid and electrolyte balance as well as systemic vascular resistance.^4^, ^5^ However, chronic activation of the RAAS results in increased glomerular filtration rate, which in turn can exacerbate proteinuria. Persistent proteinuria further damages nephrons and contributes to progression of kidney disease.^6^, ^7^ Treatment of proteinuria includes treatment of underlying diseases when present, as well as immunosuppression, if warranted. Feeding a diet supplemented with omega‐3 fatty acids, antithrombotic treatment and medications that decrease the vasoconstrictive effect of angiotensin II on the efferent glomerular arteriole system, such as angiotensin‐converting enzyme inhibitors (ACEi), angiotensin receptor blockers (ARBs), or aldosterone antagonists, also are recommended.^1^, ^8^, ^9^

Angiotensin‐converting enzyme inhibitors, such as benazepril or enalapril, have been used in veterinary medicine to treat systemic hypertension and proteinuria.^7^, ^10^, ^11^ Recently, ARBs, such as telmisartan and losartan, have been used to treat systemic hypertension in cats and dogs .^6^, ^11^, ^12^ Losartan has been used in dogs,^13^ but dogs seem to be unable to metabolize it to the active metabolite that is responsible for much of the therapeutic effect in people.^14^ By selectively blocking the angiotensin II type 1 receptor (AT1) receptor, ARBs allow the angiotensin II type 2 receptor (AT2) to remain available for activation by angiotensin II. The AT2 receptors appear to have renoprotective effects such as vasodilatation, natriuresis, and inhibition of inappropriate cell growth. Therefore, specific AT1 blockade could offer potential benefits.^15^ A meta‐analysis of 20 reports of randomized trials including over 25 000 human patients identified significant improvement in proteinuria and albuminuria as well as prevention of disease progression with telmisartan use.^16^ Two recent prospective, multicenter, placebo‐controlled blinded studies showed the safety and efficacy of telmisartan in hypertensive cats,^6^, ^12^ and a case report of refractory proteinuria in a dog showed successful treatment after introduction of telmisartan.^17^ Recently, a double‐masked randomized clinical trial indicated the efficacy of telmisartan in proteinuric dogs with chronic kidney disease, including a more rapid response compared with enalapril.^18^ Limited data, however, are available on use of telmisartan in dogs.

Our primary objective was to describe the use of telmisartan to treat proteinuria in dogs, with the hypothesis that a decrease in the urine protein‐to‐creatinine ratio (UPC) would be observed after treatment.

## 2 MATERIALS AND METHODS

### 2.1 Case selection

**FIGURE 1:**
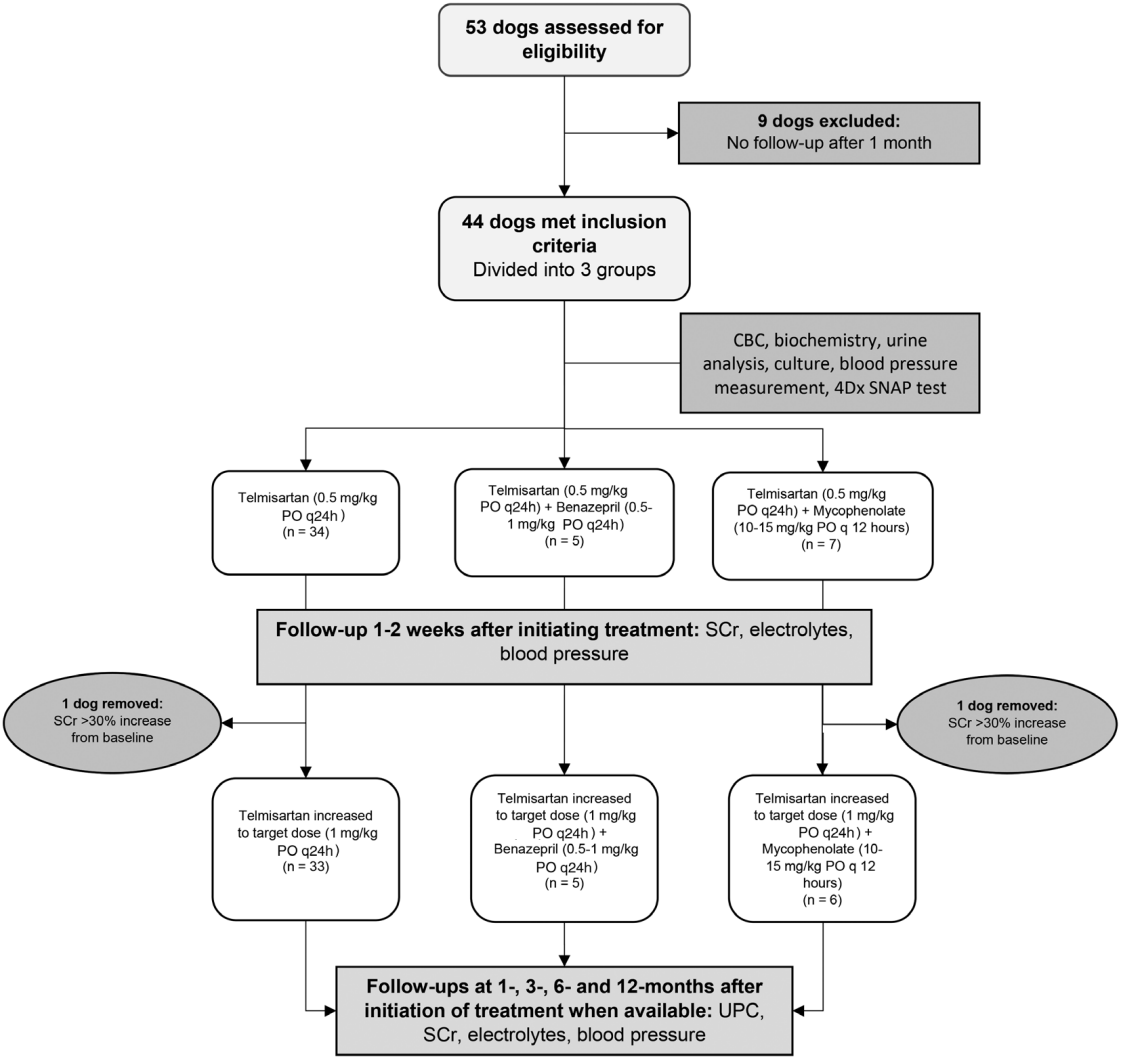
Flow diagram illustrating case selection and overview of study

Our study was a retrospective study. Electronic medical records of dogs presented at a private referral hospital (DMV Veterinary Center, Montreal, Canada) between 2017 and 2019 were reviewed. Dogs diagnosed with clinically relevant proteinuria that required treatment were included in the study. Dogs were divided into 3 groups depending on the treatment received: telmisartan alone, telmisartan with an ACEi (benazepril), and telmisartan with mycophenolate (Figure 1). The American College of Veterinary Internal Medicine (ACVIM) consensus statement guidelines were used to determine which dogs needed treatment (ie, dogs with a persistent UPC > 0.5).^1^, ^9^ Persistent proteinuria is defined as abnormal UPC on ≥3 occasions, ≥2 weeks apart.^1^ Dogs that did not have ≥3 samples before referral had their UPC re‐evaluated within 2 to 3 weeks before to the start of treatment. All dogs included in the study had hematology, serum biochemistry, urinalysis, urine culture, blood pressure measurement, and 4Dx SNAP test (Idexx Laboratories, Montreal, Canada) performed. Dogs were excluded if the 4Dx SNAP test was positive, pyuria (>6‐8 white blood cells/high power field) or bacteriuria was present on urine sediment examination or if positive urine culture results were obtained. Abdominal ultrasound examination and thoracic radiography were not required for inclusion in the study. Renal biopsies were not performed in any of the dogs.

Telmisartan was started at a dosage of 0.5 mg/kg PO q24h in all dogs. One to 2 weeks after initiating treatment, serum creatinine concentration (SCr), serum electrolyte concentrations, and systemic blood pressure were re‐evaluated. Telmisartan was increased to a target dosage of 1 mg/kg PO q24h if it was well tolerated by the dog and laboratory results remained within acceptable limits (<30% increase in SCr from baseline, serum potassium concentration < 6 mmol/L, and systolic blood pressure > 120 mm Hg).^17^ As for benazepril and mycophenolate, recommended dosages were used (0.5‐1 mg/kg PO q24h for benazepril and 10‐15 mg/kg PO q12h for mycophenolate).^19^

Adjustments were made based on the ACVIM Consensus Statement Recommendations for Standard Therapy of Glomerular Disease in dogs.^9^ Urine protein‐to‐creatinine ratio was determined before treatment and then at subsequent follow‐ups as available (1, 3, 6, and 12 months after beginning treatment). When possible, pooled urine samples were used for UPC determination, but otherwise a voided sample or samples collected by cystocentesis in the hospital were used. Samples were submitted to Idexx Laboratories for analysis (Idexx Laboratories; Miditron Junior II Urine Analyzer, Roche Diagnostics, Basel, Switzerland; Biochemical Analyzer AU680 Beckman Coulter, Brea, California). Serum creatinine and potassium concentrations also were measured (Idexx Laboratories, Biochemical Analyzer Beckman Coulter AU680, Brea, California). Systolic blood pressure was measured when possible using either a Pet Map (pet MAP graphic II, Ramsey Medical Inc, Tampa, Florida) or Doppler system (Ultrasonic Doppler Flow Detector Model 811‐B, Parks Medical Electronics Inc, Aloha, Oregon). The ACVIM Hypertension Consensus Panel and International Renal Interest Society (IRIS) guidelines and categories by target organ damage (TOD) were followed.^11^ Response to treatment was rated as complete (UPC ˂0.5), partial (UPC ≥0.5 but decreased by ≥50%), or no response (UPC decreased by <50%). Treatment protocol and whether telmisartan was used alone or in combination with another drug, was clinician‐dependent and influenced by the clinical presentation. Dogs were required to have follow‐up of their UPC at least 1 month after the start of treatment to be included in the study. Breed, age, sex, concurrent conditions, and concurrent medications were recorded for each dog. Adverse effects, as reported by the owners, were defined according to product prescribing information (ie, diarrhea, vomiting, lack of appetite, and decreased activity level).^20^ Dogs concurrently receiving corticosteroids were included; dogs diagnosed with neoplasia were excluded.

### 2.2 Data analysis

Descriptive data analysis was performed using commercially available software (Microsoft 365 Excel Data Analysis). Response to treatment is presented as percentage (%) and evaluated variables (UPC, SCr, and serum potassium concentration) are presented as mean ± SD.

## 3 RESULTS

### 3.1 Dog population

Forty‐four dogs met the inclusion criteria for the study; 9 were excluded because no follow‐up was available 1 month after the initiation of the treatment. Of the excluded dogs, 2 were euthanized (1 with atlantoaxial instability and 1 with liver dysfunction); 1 dog developed diarrhea and the owner declined subsequent follow‐up; 2 dogs developed clinically relevant azotemia before 1‐month follow‐up and treatment was discontinued. One dog died at home of unknown causes (no necropsy was performed); 2 dogs were not returned for follow‐up because of owner financial constraints and 1 dog was excluded because it tested positive for Lyme disease and rapidly improved after treatment with doxycycline. Of the 44 included dogs, 23/44 (52%) were castrated males, 17/44 (39%) spayed females, and 4/44 (9%) were intact males. Breeds represented included Yorkshire Terrier (7), mixed breed (6), miniature Schnauzer (4), Labrador Retriever (4), Chihuahua (3), Shetland Sheepdog (3), Maltese (2), Golden Retriever (2), Fox Terrier (2), miniature Poodle (1), Basset Hound (1), Newfoundland (1), Whippet (1), Doberman (1), West Highland White Terrier (1), Boston Terrier (1), Australian Shepherd (1), Portuguese Water Dog (1), Airedale Terrier (1), and American Cocker Spaniel (1). The most common concurrent conditions were hyperadrenocorticism (9), hypertriglyceridemia (6), and protein‐losing enteropathy (4). Concurrent medications received included trilostane, levothyroxine, bezafibrate, and cyanocobalamin. Of the 9 dogs being treated for hyperadrenocorticism, 6 were receiving telmisartan, 2 were receiving telmisartan with benazepril, and 1 was receiving telmisartan with mycophenolate. Of the 6 dogs being treated for hypertriglyceridemia, 4 were receiving telmisartan, and 1 each telmisartan with benazepril and telmisartan with mycophenolate. Of the 4 dogs being treated for protein‐losing enteropathy, 3 were receiving telmisartan, and 1 received telmisartan with mycophenolate. Of the dogs included in the study, 6 were being fed a commercial renal diet and 14 were receiving a polyunsaturated fatty acid supplement.

Thirty‐four dogs (34/44; 77%) also had diagnostic imaging performed (abdominal ultrasound examination and thoracic radiography). In the remaining 10 dogs, 4 (4/44; 9%) had abdominal ultrasound examination performed but no thoracic radiography, and 6 (6/44; 14%) did not have an abdominal ultrasound examination or thoracic radiography performed.

### 3.2 Urine protein‐to‐creatinine ratio

#### 3.2.1 Telmisartan

**FIGURE 2:**
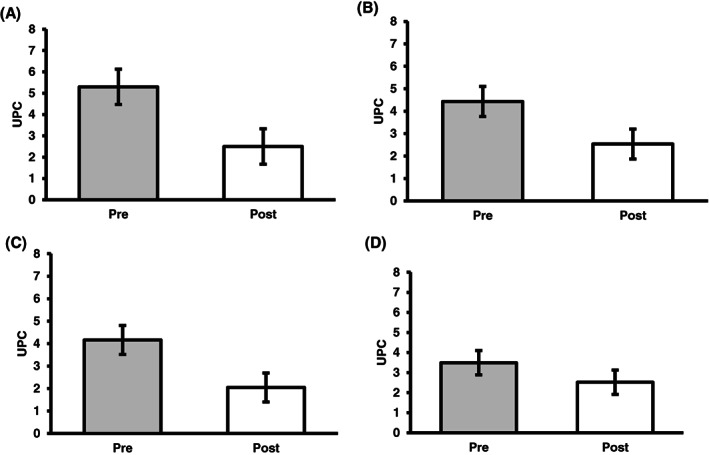
Mean UPC values in dogs being treated with telmisartan alone at 1 month (33 dogs) (A), 3 months (19 dogs) (B), 6 months (15 dogs) (C), and 12 months (10 dogs) (D) post‐treatment. Mean baseline values compared with mean post‐treatment values. UPC, urine protein : creatinine ratio

**FIGURE 3:**
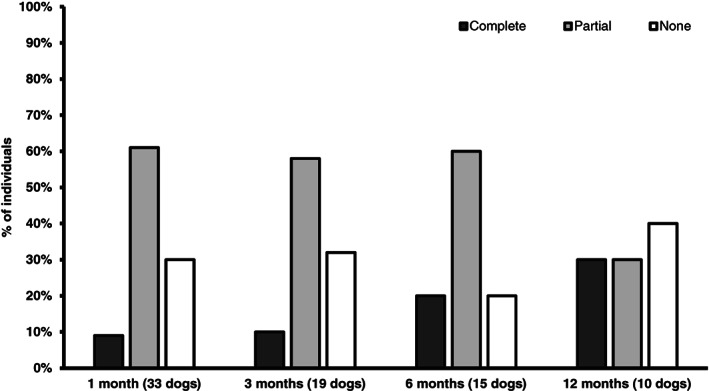
Percentage of dogs with complete (UPC ≥50% decrease from baseline but ≥0.5) (light gray bars) and no response (UPC decrease >50% from baseline) (white bars) after receiving telmisartan at 1‐, 3‐, 6‐, and 12‐month follow‐ups. UPC, urine protein : creatinine ratio

Of the 44 dogs in the study, 33 (75%) were in the telmisartan group at the 1‐month evaluation. Because the study was not standardized, not all dogs were evaluated at every follow‐up. Six (14%) dogs were evaluated at all 4 consecutive times points; 33 (75%) dogs were evaluated at 3 time points and 5 (11%) were evaluated at 2 time points. Three dogs (9%) had complete response (UPC <0.5); 20 dogs (61%) had partial response (UPC decrease ≥50% from baseline); and 10 dogs (30%) had no response (UPC decrease <50% from baseline). The mean ± SD UPC pretreatment was 5.3 ± 3.42 and it was 2.5 ± 1.22 1‐month post‐treatment (53% decrease; Figure 2A). At the 3‐month evaluation, 19 dogs (43%) were included. Two dogs (11%) had a complete response, 11 dogs (58%) had a partial response, and 6 dogs (32%) had no response. The mean ± SD UPC pretreatment was 4.43 ± 2.91 and the mean ± SD UPC 3 months post‐treatment was 2.5 ± 0.59 (43% decrease; Figure 2B). Fifteen dogs (34%) were evaluated 6 months after starting treatment. Three of these dogs (20%) had a complete response, 9 dogs (60%) had >50% decrease in UPC, and 3 dogs (20%) had no response. Mean ± SD pretreatment UPC was 4.16 ± 2.49 and mean ± SD post‐treatment UPC was 2.5 ± 0.68 (40% decrease; Figure 2C). Ten dogs (23%) had follow‐up information available 1 year after beginning treatment: 3 dogs (30%) had a complete response, 3 (30%) had a >50% decrease compared to baseline UPC, and 4 (40%) had no response. Mean ± SD pretreatment UPC was 3.49 ± 1.91 and mean ± SD UPC post‐treatment was 2.52 ± 2.70 (28% decrease; Figure 2D). The average decrease in UPC over the 12‐month period was 41% from baseline in this group. After 1 month, 70% of dogs showed a favorable response to treatment, with 9% having a complete response and 61% a partial a response. Results were similar at subsequent follow‐ups with 68%, 80%, and 60% of dogs showing response to treatment at 3‐, 6‐, and 12‐month follow‐ups, respectively (Figure 3).

#### 3.2.2 Telmisartan and benazepril

Of the 44 dogs in the study, 5 dogs (11%) received telmisartan combined with benazepril. At the 1‐month follow‐up, 1 dog had a complete response, 1 had a partial response, and 3 (60%) had no response. The mean ± SD UPC pretreatment was 3.62 ± 1.72 compared to 2.5 ± 1.35 at 1 month post‐treatment (31% decrease). At the 3‐month follow‐up, 2 dogs were included; both had no response (UPC decrease >50% from baseline). The mean ± SD pretreatment UPC was 5.0 ± 0.99 and the post‐treatment UPC was 4.4 ± 0.42. There were 3 dogs (60%) at 6 months; 2 had complete response and 1 had no response. The mean ± SD pretreatment UPC was 3.16 ± 2.27 and the mean ± SD post‐treatment UPC was 2.76 ± 4.27. Two dogs were included at 12 months; 1 had a complete response and 1 had no response; mean ± SD UPC pretreatment value was 3.4 ± 1.27 and mean ± SD UPC post‐treatment was 2.45 ± 2.89.

Of the dogs in this group, 3 of 5 were receiving telmisartan before introduction of benazepril. One dog had received telmisartan for 6 months and the UPC continued to increase during treatment (5.7 pretreatment, 4.2 after 1 month, 4.7 after 3 months, 7.7 after 6 months), which resulted in the addition of benazepril. This dog subsequently was lost to follow‐up. The second dog had an incomplete response to telmisartan after 3 months; the pretreatment UPC was 4.3 and it was 2.8 after 3 months of treatment. Benazepril was started, but all treatment was discontinued shortly thereafter because of development of unidentified liver dysfunction; no further investigation was performed. The third dog showed no response to telmisartan, and benazepril was added after 1 month; the UPC did not improve over time with combination treatment.

The remaining 2 dogs in this group had previously received benazepril. The first had been receiving benazepril for 3 months and telmisartan was introduced because of lack of improvement in the UPC (1.3). Complete therapeutic success was noted 1 month after adding telmisartan (UPC, 0.2 vs 1.3). The second dog had been receiving benazepril for 15 months, and a partial response was observed 1 month after introduction of telmisartan (UPC, 0.7 vs 2.5) and improvement continued at subsequent follow‐ups until complete response was achieved after 6 months (UPC, <0.5).

#### 3.2.3 Telmisartan and mycophenolate

Of the 44 dogs in the study, 6 (14%) received telmisartan combined with mycophenolate at the start of treatment. At the 1‐month follow‐up, 3 (50%) had a complete response, 2 (33%) had a partial response, and 1 had no response. Mean ± SD UPC pretreatment was 9.23 ± 6.93 and was 1.85 ± 1.07 1 month post‐treatment (80% decrease). At the 3‐month evaluation, 3 dogs (50%) were included. One dog had a complete response, 1 had a partial response, and 1 had no response. Pretreatment mean ± SD UPC was 13.36 ± 5.92 and was 1.46 ± 1.94 post‐treatment (89% decrease). At the 6‐month evaluation, 3 dogs (50%) were included. Two dogs had a complete response and 1 dog had no response. The mean ± SD UPC pretreatment was 13.36 ± 5.92 and was 1.53 ± 2.31 post‐treatment (89% decrease). At the 12‐month evaluation, 3 dogs were included; 2 had a complete response and 1 dog had a partial response. The pretreatment mean ± SD UPC was 13.36 ± 5.92 and post‐treatment was 1.1 ± 1.47 (92% decrease).

### 3.3 Serum creatinine concentration

Six dogs (14%) had azotemia before the start of treatment (SCr range, 1.4‐2.8 mg/dL). Thirty‐three dogs (75%) had SCr measured after 1 month: mean ± SD pretreatment SCr was 1.06 ± 0.5 mg/dL and was 1.13 ± 0.46 mg/dL (reference range, 0.5‐1.5 mg/dL) post‐treatment (<30% increase). Twenty‐one dogs (48%) were evaluated after 3 months; pretreatment mean ± SD SCr was 1.02 ± 0.38 mg/dL and was 1.06 ± 0.39 mg/dL post‐treatment (<30% increase). Twenty dogs (46%) were evaluated after 6 months; mean ± SD pretreatment SCr was 1.04 ± 0.41 mg/dL and was 1.12 ± 0.49 mg/dL post‐treatment (<30% increase). At the 12‐month evaluation, 15 dogs (15/44; 34%) were evaluated. The pretreatment mean ± SD SCr was 1.11 ± 0.62 mg/dL, and after treatment, was 1.29 ± 0.59 mg/dL (<30% increase). Two dogs developed clinically relevant azotemia before the 1‐month follow‐up and were excluded from the study. The first dog was in the telmisartan group and SCr increased to 3.22 mg/dL (from 0.87 mg/dL). The second dog was in the telmisartan and mycophenolate group and SCr increased to 2.84 mg/dL (from 0.88 mg/dL). The first dog was lost at follow‐up and the second subsequently was euthanized because of deterioration in overall condition.

### 3.4 Serum potassium concentration

Thirty‐three dogs (75%) had serum potassium concentration evaluated after 1 month. The pretreatment mean ± SD serum potassium concentration was 4.64 ± 0.47 mmol/L and was 4.61 ± 0.45 mmol/L post‐treatment (reference range, 4.0‐5.4 mmol/L). Twenty‐one dogs (48%) were evaluated after 3 months; the pretreatment mean ± SD serum potassium concentration was 4.56 ± 0.55 mmol/L and was 4.95 ± 0.41 mmol/L post‐treatment. Twenty dogs (46%) were evaluated after 6 months; the mean ± SD pretreatment serum potassium concentration was 4.59 ± 0.73 mmol/L and was 4.7 ± 0.62 mmol/L post‐treatment. At the 12‐month evaluation, 15 dogs (34%) were evaluated. The pretreatment mean ± SD serum potassium concentration was 4.65 ± 0.82 mmol/L and after treatment was 4.57 ± 0.77 mmol/L. No dog had a serum potassium concentration > 6 mmol/L at any time.

### 3.5 Blood pressure

**FIGURE 4:**
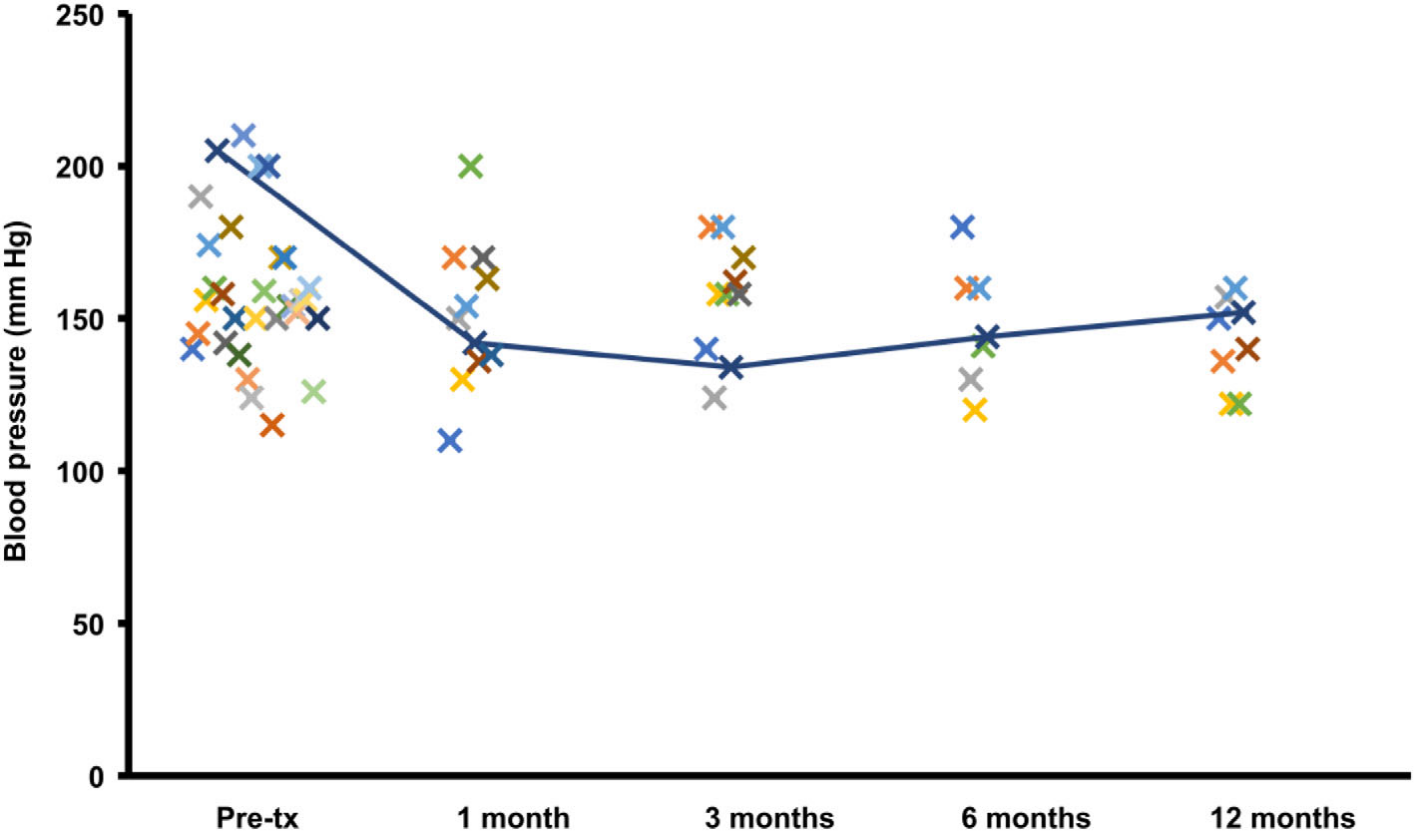
Systemic blood pressure measurements over time at 1, 3, 6, and 12 month in dogs being treated with telmisartan for proteinuria

Thirty‐one dogs had their blood pressure evaluated and recorded before treatment; the average blood pressure was 159 mm Hg (range, 115‐205 mm Hg). Nine dogs (21%) had blood pressure evaluated after 1 month. Their pretreatment mean blood pressure was 165 mm Hg and was 154 mm Hg post‐treatment. Ten dogs (23%) were evaluated after 3 months; mean pretreatment blood pressure was 162 mm Hg and was 156 mm Hg post‐treatment. Six dogs (14%) were evaluated after 6 months; mean pretreatment blood pressure was 172 mm Hg and was 149 mm Hg post‐treatment. At the 12‐month evaluation, 6 dogs (14%) were evaluated; mean pretreatment blood pressure was 152 mm Hg and after treatment was 142 mm Hg (Figure 4). No dogs in the study received amlodipine.

### 3.6 Average dosage used

The average telmisartan dosage used was 0.92 mg/kg PO q24h (range, 0.34‐1.45 mg/kg). The 2 dogs excluded because of azotemia were receiving dosages of 0.34 mg/kg and 0.5 mg/kg, respectively. The average dosage for benazepril was 0.67 mg/kg PO q24h (range, 0.47‐1.28 mg/kg) and the average dosage for mycophenolate was 11.47 mg/kg PO q12h (range, 10.2‐13.3 mg/kg).

### 3.7 Adverse effects

Of 44 dogs, 5 (11%) were reported to have adverse effects after introduction of telmisartan. These consisted of mild gastrointestinal signs (anorexia and diarrhea), which were self‐limiting. Two dogs developed clinically relevant azotemia (increase in SCr >30%) that required discontinuation of the treatment before the 1‐month follow‐up; these dogs subsequently were excluded.

## 4 DISCUSSION

The purpose of our retrospective study was to describe the use of telmisartan in dogs to treat proteinuria in various clinical situations. A previous study in cats with chronic kidney disease showed that telmisartan was at least as effective as benazepril and significantly decreased proteinuria after treatment, and a recently published clinical trial on telmisartan use in dogs showed similar results in comparison with enalapril.^18^, ^20^ Our study also showed a substantial decrease in proteinuria compared to baseline in dogs treated with telmisartan.

In the group of dogs treated only with telmisartan, most showed a therapeutic response over the course of the study, and a post‐treatment UPC <0.5 or a decrease of ≥50% from baseline.^9^ Some dogs showed complete response early in the course of treatment whereas others achieved a response later on. At the 1‐month follow‐up, 70% of dogs showed a favorable response, with 9% having a complete response and 61% having a partial response. Results were similar at subsequent follow‐ups, with 68%, 80%, and 60% of dogs showing favorable response to treatment at 3‐, 6‐, and 12‐month follow‐ups, respectively. The average decrease over the study period was 41%, which is similar to what is reported in human medical literature. Human patients experienced an average decrease of 35% in UPC over a 3.4‐year period, but most of the decrease occurred in the first 6 months of treatment.^21^, ^22^ Most improvement seems to occur after treatment is first introduced, and that the UPC results then remain stable over time. A prospective study of longer duration would be needed to investigate if this trend also applies to dogs.

In the dogs that received both telmisartan and benazepril, 1 dog achieved complete response even though the dog had failed to show a clinically relevant response after receiving benazepril for the previous 15 months. Because this dog had been treated with an ACEi for a long period of time, an ACE escape mechanism (alternate pathway for angiotensin II generation) could have prevented further response before introduction of an ARB.^23^ The ARBs are not affected by the ACE escape mechanism, which may explain the continued decrease in proteinuria in this animal.^20^ Over time, inhibition of angiotensin converting enzyme decreases with the use of ACEi, allowing plasma concentrations of angiotensin II to increase. This could explain why higher doses of ACEi are sometimes needed and why combination with an ARB can improve the therapeutic response.^24^

The 3 dogs that previously received telmisartan failed to show improvement when benazepril was added as combination treatment. Because specific receptor blockade by telmisartan already was present, the added inhibition of angiotensin converting enzyme by benazepril might have failed to provide further blockade and hence the lack of response. Combination treatment with an ARB and an ACEi is thought to offer more complete blockade of the RAAS compared to monotherapy, and studies in people have suggested a potential combined effect in treating proteinuria.^21^, ^22^, ^25^, ^26^ A meta‐analysis of people with diabetic nephropathy showed that combination treatment significantly decreased proteinuria compared to monotherapy.^27^ However, more recently, studies in people have found that optimal RAAS blockade may be desired over complete blockade, thus minimizing potential adverse effects encountered with complete blockade commonly observed with combined treatment.^20^, ^21^, ^22^, ^23^, ^24^, ^25^, ^26^, ^27^, ^28^, ^29^, ^30^, ^31^ A recently published clinical trial in dogs showed that 31% of dogs developed significant increases in SCr requiring hospitalization for treatment, further supporting this argument.^18^ Blockade at the angiotensin‐II receptors by ARBs could offer a potential benefit over the use of ACEi, especially in dogs refractory to treatment, as was observed in a recent clinical trial.^18^ We also cannot exclude that the 2 dogs in our study that had complete response with combination treatment after introduction of telmisartan could have had a similar response had the ACEi been gradually withdrawn or discontinued, as previously described in a case report.^17^

Six dogs were treated with both telmisartan and mycophenolate, and a partial or complete response was observed for most of these dogs at the follow‐up visits. This combined treatment was generally well tolerated with no clinically important adverse effects reported for dogs in this group. Although a potential underlying immune‐mediated process was suspected in these cases, no specific UPC result indicates a specific renal disease.^32^ A recent study evaluating dogs with focal segmental glomerulosclerosis confirmed by histopathology showed a median UPC of 5.9 with UPCs ranging from 1.4 to 22, and suggesting that the severity of proteinuria cannot be correlated with an immune‐mediated cause.^33^ Although an immunosuppressive treatment protocol has yet to be thoroughly investigated in dogs, treatment is recommended in cases in which biopsy results are suggestive of immunopathogenesis, or if azotemia is progressive, severe hypoalbuminemia is present and a lack of response to standard treatment occurs in the absence of a histopathological diagnosis.^34^, ^35^, ^36^, ^37^

The use of the immunosuppressive drug mycophenolate in our study might have been responsible for the positive response observed in these dogs. However, a previous prospective clinical trial did show a favorable response in dogs treated with enalapril for idiopathic glomerulonephritis.^10^ It is therefore possible that telmisartan could provide a similar effect. Renal biopsies were not performed in our study to confirm the clinical suspicion of immunopathogenesis. Although biopsy enables the clinician to confirm the pathology present,^34^ this procedure is not commonly performed in our private practice. The decision to combine immunosuppressive treatment in combination with telmisartan was clinician‐dependent and based on the severity of the proteinuria and lack of known underlying cause.^35^ A larger scale study with a control group and renal histopathology results would be required to draw pertinent conclusions regarding this particular group.

Serum creatinine concentrations remained within the reference range for all dogs except for 2 that developed clinically relevant azotemia (>30% increase in SCr) shortly after starting treatment, which resulted in treatment cessation. In the remainder of dogs evaluated, all increases in SCr were <30% and treatment could be continued according to accepted guidelines.^9^ Because blockade of the RAAS may cause a decrease in glomerular filtration rate, acute azotemia could be a potential adverse effect of telmisartan use, similar to what is observed with ACEi use.^38^, ^39^ For that reason, we routinely recommend follow‐up 1 to 2 weeks after initiation of treatment to ensure it is well tolerated by the dog and before increasing the dose into the target range.

In our study, a small percentage of dogs (5/44; 11%) also developed some mild gastrointestinal signs after introduction of telmisartan. These signs were self‐limiting and did not preclude continuation of treatment. These signs included anorexia and diarrhea, which also have been reported in cats.^19^ Although the clinical signs appeared shortly after introducing telmisartan, we cannot completely exclude the possibility of another cause for these clinical signs such as primary gastrointestinal disease or concurrent medications.

Serum potassium concentration also was evaluated, and concentrations remained within the normal reference range for all dogs. No dog had a serum potassium concentration > 6 mmol/L.^9^ Studies in human patients suggest that ARBs are less likely than ACEi to cause hyperkalemia because they cause a smaller decrease in plasma aldosterone concentration. Different hypotheses have been made to explain the possible mechanisms involved, such as an effect on glomerular filtration rate, tissue penetrability, and various hormones that could affect serum potassium concentration.^40^, ^41^ In a recently published clinical trial, however, no significant difference was noted when comparing percentage change relative to baseline in SCr and serum potassium concentration when comparing dogs being treated with telmisartan and to those treated with enalapril.^18^

Thirty‐one dogs (71%) had their blood pressure evaluated before treatment, and mean systolic blood pressure was in the low TOD category (140‐159 mm Hg).^11^ Blood pressure remained in an acceptable range (>120 mm Hg)^18^ for all dogs at the various follow‐ups. Not all dogs had blood pressure measurements noted in their records at each follow‐up for different reasons (eg, lack of dog compliance, financial restrictions, incomplete medical records). Telmisartan is approved for treatment of hypertension in cats^6^ and its use in human patients also has shown it to be an effective antihypertensive agent.^42^ A clinical trial evaluating the efficacy of telmisartan in dogs with renal proteinuria showed a more significant decrease in systolic blood pressure compared to dogs treated with enalapril.^18^ Additional studies would be necessary to evaluate if telmisartan also could be used to treat hypertension in dogs.

Our study had several limitations because of its retrospective nature. Not all dogs had complete standardized diagnostic evaluations (no imaging performed in some dogs and no renal biopsies were performed) and therefore potential underlying causes of proteinuria may have been missed. Some dogs were not evaluated at each recommended follow‐up interval, which could have affected overall results. Method of urine collection varied among dogs. Previous studies have shown that, for most dogs, overall results are similar regardless of the method used, but samples collected in the hospital could yield higher results.^43^ Different devices were used to evaluate systemic blood pressure which could have introduced substantial variability. Furthermore, because many dogs had various concurrent diseases, these could have affected the severity of proteinuria.^1^ Some dogs also were receiving concurrent medications, special diets and supplements to manage these conditions and could have shown improvement over time as their medical condition was better controlled. Some dogs also were being treated concurrently for protein‐losing enteropathy and required corticosteroid treatment that also could have affected the extent of proteinuria.

The aim of our study was to describe the use of telmisartan for treatment of proteinuria in dogs in various clinical situations. Our results show that most dogs experienced a decrease in the severity of proteinuria after initiation of telmisartan and the drug overall was well tolerated. Because of the different disease processes present in the dogs and different concurrent treatments, the decrease in proteinuria cannot be attributed to telmisartan alone for all dogs. Additional standardized prospective studies are needed to further validate our findings.

## CONFLICT OF INTEREST DECLARATION

Authors declare no conflict of interest.

## OFF‐LABEL ANTIMICROBIAL DECLARATION

Authors declare no off‐label use of antimicrobials.

## INSTITUTIONAL ANIMAL CARE AND USE COMMITTEE (IACUC) OR OTHER APPROVAL DECLARATION

Authors declare no IACUC or other approval was needed.

## HUMAN ETHICS APPROVAL DECLARATION

Authors declare human ethics approval was not needed for this study.
